# Short or Long Antibiotic Regimes in Orthopaedics (SOLARIO): a randomised controlled open-label non-inferiority trial of duration of systemic antibiotics in adults with orthopaedic infection treated operatively with local antibiotic therapy

**DOI:** 10.1186/s13063-019-3832-3

**Published:** 2019-12-09

**Authors:** Maria Dudareva, Michelle Kümin, Werner Vach, Klaus Kaier, Jamie Ferguson, Martin McNally, Matthew Scarborough

**Affiliations:** 1 Bone Infection Unit, Nuffield Orthopaedic Centre, Oxford University Hospitals, Oxford, UK; 2 Nuffield Department of Medicine, University of Oxford, Oxford, UK; 3 Department of Orthopaedics and Traumatology, Universitätsspital Basel, Basel, Switzerland; 4 Institute of Medical Biometry and Medical Informatics, Universitätsklinikums Freiburg, Freiburg, Germany

**Keywords:** Osteomyelitis, Prosthetic joint infection, Diabetic foot, Antibiotic, Duration, Carrier, Revision, Local antibiotic therapy

## Abstract

**Background:**

Orthopaedic infections, such as osteomyelitis, diabetic foot infection and prosthetic joint infection, are most commonly treated by a combination of surgical debridement and a prolonged course of systemic antibiotics, usually for at least 4–6 weeks. Use of local antibiotics, implanted directly into the site of infection at the time of surgery, may improve antibiotic delivery and allow us to shorten the duration of systemic antibiotic therapy, thereby limiting the frequency of side effects, cost and selection pressure for antimicrobial resistance.

**Methods:**

SOLARIO is a multicentre open-label randomised controlled non-inferiority trial comparing short and long systemic antibiotic therapy alongside local antibiotic therapy. Adult patients with orthopaedic infection, who have given informed consent, will be eligible to participate in the study provided that no micro-organisms identified from deep tissue samples are resistant to locally implanted antibiotics. Participants will be randomised in a 1:1 ratio to receive either a short course (≤ 7 days) or currently recommended long course (≥ 4 weeks) of systemic antibiotics. The primary outcome will be treatment failure by 12 months after surgery, as ascertained by an independent Endpoint Committee blinded to treatment allocation. An absolute non-inferiority margin of 10% will be used for both per-protocol and intention-to-treat populations. Secondary outcomes will include probable and definite treatment failure, serious adverse events, treatment side effects, quality of life scores and cost analysis.

**Discussion:**

This study aims to assess a treatment strategy that may enable the reduction of systemic antibiotic use for patients with orthopaedic infection. If this strategy is non-inferior, this will be to the advantage of patients and contribute to antimicrobial stewardship.

**Trial registration:**

Clinicaltrials.gov, NCT03806166. Registered on 11 November 2019.

## Background

The overuse of antibiotics contributes to healthcare costs, adverse drug reactions and the rising threat of antibiotic resistance. In 2016, antibiotic use in England alone cost > £200 million [1]. Around one in six patients treated with antibiotics for chronic osteomyelitis experience adverse drug reactions [2, 3] and bacterial resistance to broad-spectrum last resort antibiotics, such as carbapenems, has risen substantially in the UK over the past 10 years, driven by evolution under selection pressure from systemic antibiotic use [4, 5]. Furthermore, exposure to systemic antibiotic therapy increases the risk of subsequent antibiotic-resistant infection [6–8]. Consequently, the recommended duration of treatment for many common infectious diseases has been reduced in recent years [4, 5].

Orthopaedic infections include osteomyelitis, prosthetic joint infection, fracture-related infection and diabetic foot infection. Treatment usually involves surgery combined with antibiotics. Systemic antibiotics alone are commonly ineffective, primarily due to the persistence of bacteria in biofilm on bone and implant surfaces. Antibiotics penetrate biofilm poorly, where bacteria additionally express a quiescent metabolic state that confers antibiotic tolerance [9]. Consequently, at least 4–6 weeks of postoperative oral or intravenous antibiotics are currently recommended for orthopaedic infections, which is much longer than for common soft-tissue bacterial infections [10–13].

Increasingly, systemic antibiotics are now being used in combination with local antibiotic therapy delivered directly to the site of orthopaedic infection at the time of surgery [14–21]. Local antibiotic therapy allows high concentrations of antibiotic to be delivered rapidly to the bone and surrounding tissue with limited risk of systemic absorption and consequent side effects [14, 15, 20, 22–25]. Modern bioabsorbable ceramic carriers are able to elute therapeutic concentrations of antibiotic for > 7 days, based on pharmacokinetic studies [26–29]. Hence, local antibiotic therapy may allow much shorter courses of systemic antibiotics to be used for orthopaedic infection [26, 30–34] but, in the absence of prospective comparison with longer courses, this approach has not been widely adopted.

Previous trials [35–37] that have compared local antibiotic therapy against systemic antibiotic treatment for orthopaedic infection have been limited by low numbers and potential bias due to deviation from the allocated treatment strategy. Many people treated for orthopaedic infection now receive local antibiotic therapy as part of routine surgical treatment which allows the comparison of standard long courses of systemic antibiotic treatment for these patients with short course treatment within routine clinical care.

## Methods

### Objectives

#### Primary objective

The primary objective of the present study is to determine whether treatment of orthopaedic infection with local antibiotics combined with ≤ 7 days of systemic therapy (oral or intravenous) is non-inferior to treatment with local antibiotics combined with ≥ 4 weeks of systemic therapy (oral or intravenous), as assessed by treatment failure rate at one year.

#### Secondary objectives

Secondary objectives of the present study are to compare the following secondary endpoints according to treatment allocation:

1. probable treatment failure and possible treatment failure;
2. serious adverse events (SAEs), including death (i.e. all-cause mortality);
3. antibiotic side effects related to the treatment of orthopaedic infection;
4. resource allocation using (a) length of inpatient hospital stay, (b) frequency of outpatient visits, (c) antibiotic prescribing costs. In-hospital treatment cost analysis;
5. quality of life as evaluated by the patient-reported outcome measures on the EQ-5D-5 L questionnaire at baseline and one-year follow-up;
6. deviation from allocated treatment strategy, including additional antibiotic prescribing, and early termination of systemic antibiotics because of AEs, patient preference or any other reason.

### Study design

SOLARIO is a parallel-group, open-label study randomising individual participants to short or long systemic antibiotic regimes, each in combination with local antibiotic therapy. The primary endpoint is definite treatment failure at 12 months. Any post-randomisation readmission or reoperation with signs or symptoms at the anatomical site of infection will be considered a potential treatment failure. An independent Endpoint Committee, blind to allocation, will review all potential treatment failures to determine whether a primary or secondary infection-related endpoint has been reached.

The choice of antimicrobial agents used will not be dictated by the trial protocol. Choice of specific agents, both local and systemic, will be as determined by the clinician caring for the patient, and will be chosen according to the available clinical and microbiological data, local epidemiology and good clinical practice.

### Participants

This study will recruit adults undergoing surgical treatment for orthopaedic infection where the surgery includes the implantation of a licensed local antibiotic-carrier combination at the site of infection.

### Study centres

This multicentre study will take place across multiple orthopaedic centres with expertise and experience of providing specialist care for orthopaedic infection in the UK and mainland Europe. A full list of active study sites is available at www.clinicaltrials.gov (NCT03806166, registered on 11 January 2019).

### Eligibility criteria

A patient must meet ALL of the following criteria to take part:

1. Provision of informed consent within seven days after surgery;
2. Aged ≥ 18 years;
3. Presenting with an orthopaedic infection as defined by one or more of the following criteria:

- localised pain, OR
- localised erythema, OR
- temperature ≥ 38.0 °C, OR
- a discharging sinus or wound;

4) Undergoing surgical treatment for the infection;
5) Locally administered licensed antibiotic-carrier combination(s) at the site of orthopaedic infection;
6) Would ordinarily be managed with a prolonged course (≥ 4 weeks) of systemic antibiotic(s);
7) Specimens for microbiological analysis taken at index surgery.

All patients included in the study will have a maximum of seven days of systemic antimicrobial therapy, as randomisation has to be within seven days.

A patient may not enter the study if ANY of the exclusion criteria listed below apply.

#### Surgical exclusion criteria

1. The index operation was not a definitive procedure with the aim of eradicating infection:
  - Primary closure has not been achieved, or
  - Re-look surgery is planned;
2. The index operation involved implant retention at the site of index infection (e.g. Debridement, Antibiotics and Implant Retention).

#### Microbiological exclusion criteria

3) Any identified micro-organisms from operative specimens from the site of index infection that are fully resistant to the local antibiotic(s) administered at the site of infection.

#### Medical exclusion criteria

4) Any other infection necessitating additional systemic antibiotic treatment > 7 days after surgery, such as *Staphylococcus aureus* bacteraemia or bacterial endocarditis;
5) If the patient is in a clinical trial involving an Investigational Medicinal Product (IMP) related to infection.

### Interventions

The intervention assessed in this study is the reduction of the duration of systemic antibiotic therapy from ≥ 4 weeks to ≤ 7 days. All patients will have local antibiotic therapy given at the time of surgery as part of their planned treatment.

### Concomitant care and interventions permitted in parallel

Clinical care for the participant, other than the duration of systemic antibiotic treatment for orthopaedic infection, will not be influenced by participation in this study. All treatment decisions will be made by the participant in partnership with the clinical care team.

### Outcomes

Endpoints will be identified by prospective surveillance throughout the year after randomisation. This study is an open-label trial, but the ascertainment of outcomes will be carried out by an endpoint committee who will be blind to the participant’s treatment allocation (blinded Endpoint Committee).

The definitions of infection recurrence used in this study are based on internationally accepted standards [9, 10, 38] with minor modifications. They have been applied in a previous study in orthopaedic infection [3].

### Primary endpoint

The primary endpoint will be definite treatment failure within 12 months of surgery, as determined by a blinded Endpoint Committee using notes from the patient records redacted for names of patients and clinicians, as well as information that might betray the allocated treatment strategy. The criteria for definite, possible and probable treatment failure are available in the Additional file 1.

### Secondary endpoints

Secondary endpoints include probable and possible treatment failure, as determined by the blinded Endpoint Committee. Definitions and information about validation for these endpoint measures are available in the Additional file 1. Additional secondary endpoints, discussed below, will be determined by the research team at individual sites.

Symptoms potentially attributable to side effects of antibiotic treatment will be recorded on a three-point scale (absent, mild, severe) for all participants, regardless of whether antibiotics are taken at the time [39]. Five potential side effects, identified as having the highest impact by a group of 16 patient representatives, will contribute to a bivariate analysis; all additional reported symptoms that may represent antibiotic-related side effects will be similarly recorded on the three-point scale and reported as proportions of participants affected.

Quality of life will be assessed using the EQ-5D-5 L questionnaire administered at study enrolment and 12 months after surgical treatment of infection. For the enrolment questionnaire, patients will be asked to describe their health state immediately before index surgery. The mean difference between quality of life scores at baseline and 12 months will be compared. Permission to use the EQ-5D-5 L questionnaire was granted by the EuroQol Research Foundation.

### Blinded Endpoint Committee

A blinded Endpoint Committee will comprise three independent clinicians with specialist training in orthopaedic surgery or clinical infection. The blinded Endpoint Committee will remain unaware of participants’ treatment allocation. Any symptoms or signs identified from the hospital notes or when the participant is reviewed at follow-up that, in the opinion of the study clinicians, may meet the definition of treatment failure, will be reported to the Committee. Additionally, the following clinical events during the trial follow-up period will be reported, so that the Endpoint Committee can assess whether an endpoint has occurred:

1. Readmission to hospital for investigation or treatment of illness that may be associated with the site of incident infection;
2. Unplanned reoperation at the anatomical site of infection;
3. Wound dehiscence, discharge or appearance of new sinus at the site of incident infection;
4. Prescription of additional systemic antibiotics for orthopaedic infection at the incident anatomic site after completion of systemic antibiotic treatment for index orthopaedic infection;
5. Mortality during the trial follow-up period;
6. Concern from the local study clinicians that there has been a recurrence of infection, where none of the above criteria have been met.

### Participant timeline

**Fig. 1:**
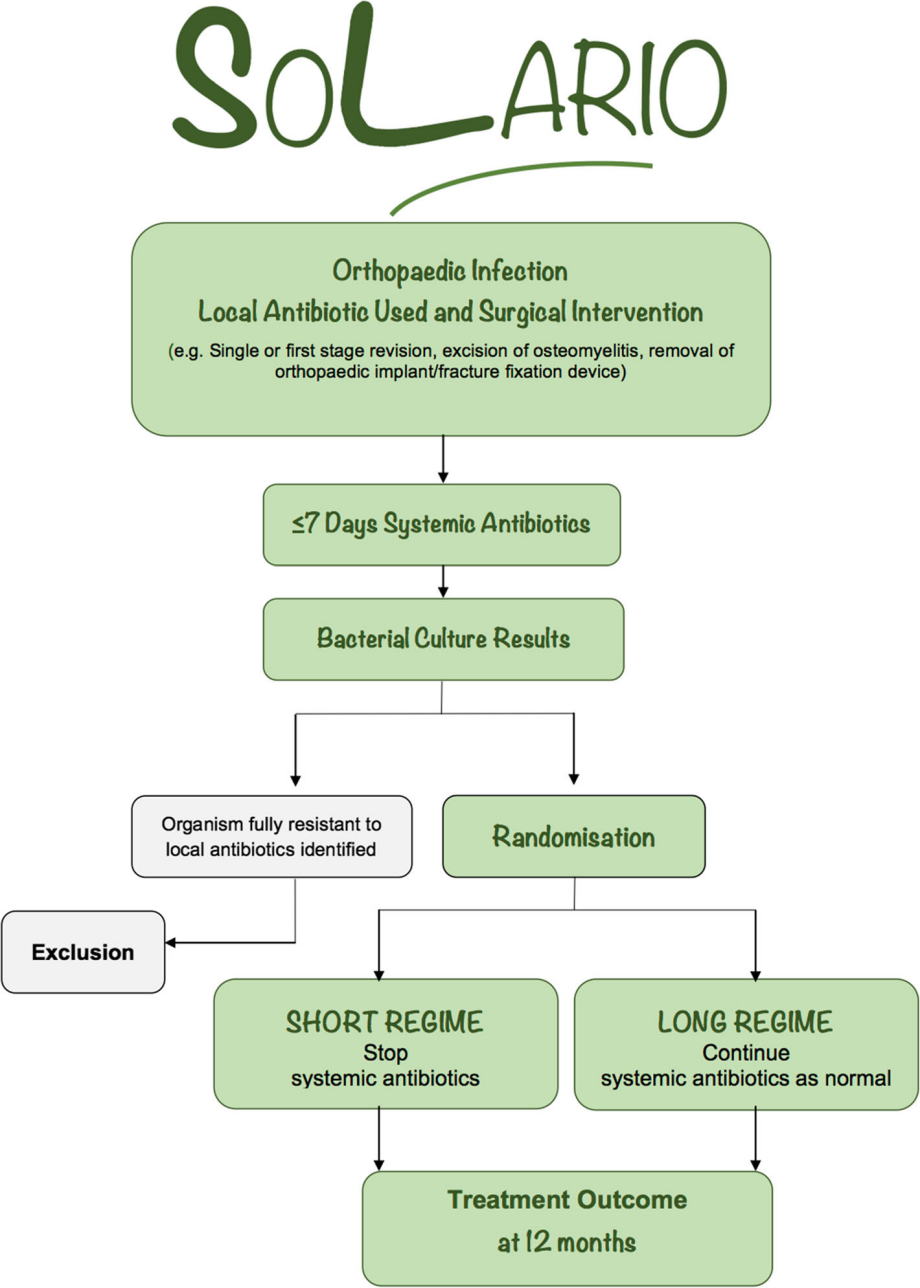
*Flow diagram* of participant enrolment, randomisation, treatment and follow-up within the SOLARIO study

**Fig. 2:**
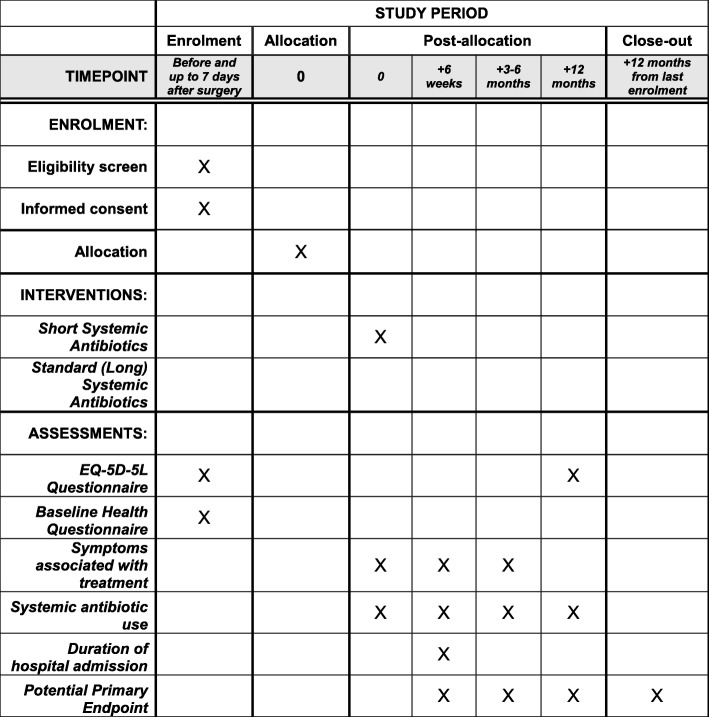
SPIRIT Schedule of enrolment, interventions and assessments in the SOLARIO study

The timeline for participants in the study is shown in Figs. 1 and 2. Adult patients referred to an infectious disease physician or orthopaedic surgeon for the treatment of orthopaedic infection, whose treatment plan includes surgical management and local antibiotic therapy, will be considered for inclusion in the study.

Local clinical research staff will seek informed consent to participate in the study from eligible patients. After receipt of the Patient Information Sheet, potential participants will be encouraged to discuss the study with the clinical care team and/or personal contacts and to ask questions. The Patient Information Sheet and Informed Consent Form state clearly that taking part in the study is voluntary and that refusal or withdrawal carry no detriment to usual clinical care. Patients not felt to have capacity to consent to study participation will not be approached and proxy consent will not be allowed. This trial does not involve any study-specific outpatient appointments, investigations or hospital visits. Hence, there will be no expenses or other payments to participants in this study.

Potential participants will be afforded up to seven days after their surgery to decide whether they wish to take part. Those that provide fully informed consent will be randomised through a centralised electronic database with stratification by study site only.

Participants will be followed up during routine care visits at the local centre. The frequency and timings of routine care visits will vary depending on the clinical condition and management pathways of individual patients. Trial-specific data collection points will be at: (1) 6 weeks (± 1 week); (2) once between 3 and 6 months; and (3) 1 year (± 1 month) after randomisation. There will be no trial-specific clinic visits. Either the patient or the patient’s GP will be asked for follow-up data if information relating to outcome measures is not available through routine care visits at these times.

### Sample size

A total of 500 participants will be recruited into this study (250 in each arm). This is based on an anticipated failure rate of up to 13% in the study population overall, 90% power to demonstrate non-inferiority with a non-inferiority margin of 10%. Corresponding simulations indicated a necessary total sample size of 492, rounded up to 500. Deviation from protocol will be accounted for through per-protocol and intention-to-treat analyses conducted in parallel for outcomes relating to infection recurrence.

### Strategies for ensuring adequate enrolment for sample size

Recruitment at the proposed centres, which were identified from a previous study of treatment strategy for orthopaedic infection as high recruiting centres that also extensively used local antibiotic therapy [3], will help 500 participants to be enrolled into the trial during a 24-month recruitment period. Additional centres will also be approached to improve recruitment.

### Assignment of interventions and allocation concealment

Participants will be allocated to short or long systemic antibiotic treatment at random in a 1:1 ratio within seven days after index surgery.

Randomisation using permuted blocks, stratified by treatment centre only, is allocated through the Clinical Database Management System (CDMS). Research enrolment and randomisation will be performed by the local clinical research staff for participants who give informed consent to participate in the study. Allocation concealment is inherent to the software used for randomisation.

Back-up offline randomisation will be available and is described in Additional file 1.

The generation of randomisation sequences is described in Additional file 1.

### Blinding

This is an open-label study; only the blinded Endpoint Committee will be unaware of the participant’s treatment allocation when assessing potential primary endpoints using redacted patient records. Information that may inadvertently disclose treatment allocation will be redacted, including antibiotic use, antibiotic side effects, antibiotic monitoring and means of antibiotic administration (including venous catheterisation).

### Data collection and management

Data will be collected through an electronic case report form within a CDMS. Validation, additional data entry instructions and scheduling will be employed within the CDMS to improve data quality and assist contemporaneous recording. Data will be stored securely and will be accessible by non-clinical research staff for authorised data monitoring purposes only. Data entry and database access will be fully logged and audited. Missing data will be sought by local study clinicians by telephone from participants or their GP if consent for this has been provided. Anonymised data without patient identifiable information will be used for analysis.

Participants whose treatment deviates from allocated treatment strategy will be followed up within the study in the same way to enable analysis by intention to treat.

### Statistical analysis

The non-inferiority margin is 10%, i.e. a tolerance of < 10 additional treatment failures for every 100 patients treated. This margin has been used in previous studies of bone infection [40]. Nine out of 16 patient representatives at the Nuffield Orthopaedic Centre, Oxford, UK, with experience of orthopaedic illness, voluntarily involved in the design of this study, preferred to accept an 8/10 chance of cure for orthopaedic infection rather than endure four weeks of emphasised severe side effects for a 9/10 chance of cure. However, 4/16 patient representatives would not accept a decrease in the chance of cure from 99% to 98% to avoid four weeks of severe antibiotic side effects, highlighting the individual nature of this decision.

For the purpose of the primary analysis, participants experiencing definite treatment failure by 12 months (as classified by the blinded Endpoint Committee) will be regarded as treatment failures and all other patients as free from treatment failure. Though the absolute rate of treatment failure may not be accurately estimated this way, the size of the risk difference between the two treatment arms is an appropriate estimate of the true difference. A formal assessment of the difference between the two arms will be based on the risk difference with a 95% confidence interval and a one-sided test (*p* = 0.025) to reject the null hypothesis that the risk difference is greater than the non-inferiority margin. Both intention-to-treat and per-protocol comparisons of the failure rate between the two treatment arms will be included as part of the primary analysis and non-inferiority will be required in both analyses to support the conclusion of short systemic antibiotic treatment non-inferiority in this study.

Further contingency analyses will compare the proportion of additional possible and probable treatment failures in both treatment arms.

Comparison of proportions of participants experiencing the primary endpoint, rather than time-to-event analysis, has been chosen because participants experiencing symptoms of infection recurrence would be very likely to seek review at their specialist centre or have information pertaining to specialist review available through their GP; the assumption of non-informative censoring would not be met.

#### Bivariate analysis

The potential advantage of short systemic antibiotics (fewer side effects) versus the potential disadvantage (higher treatment failure rate) will be summarised at the patient level in an advantage score.

The advantage score is defined as the sum of predefined side effect scores at the first two follow-up timepoints: a 1-point increase is equivalent to, in either follow-up period, one level higher for one of each of the five predefined side effects or the presence of either *Clostridium difficile* diarrhoea or intravenous catheter-associated complication. A formal assessment of the difference between the two arms with respect to the advantage score will be based on the difference in mean values with a 95% confidence interval and a *p* value for the test of the null hypothesis of no difference between the treatment groups.

In addition, the difference in mean advantage score and the difference in treatment failure rate will be plotted in a two-dimensional plane together with a 95% confidence region. Furthermore, the ration between the scores will be reported together with a 95% confidence interval. This will allow assessment of the improvement in mean advantage score required for one more treatment failure in 100 patients in order to regard short systemic antibiotic treatment as beneficial. Further secondary analyses are described in Additional file 1.

Data monitoring, interim analysis and criteria for early discontinuation of the study are described in Additional file 1.

## Discussion

All investigators involved in the study have acknowledged a position of equipoise in relation to treatment for bone and joint infections; they accept that there is currently insufficient robust clinical evidence defining the optimal duration of systemic antibiotics for management of bone and joint infection.

This study aims to compare the alternative strategies of short and long systemic antibiotic therapy for patients already receiving local antibiotic therapy as part of their standard care for orthopaedic infection. It is recognised that there is heterogeneity within the presenting orthopaedic infection, the local antibiotic therapy used (including antibiotic and carrier properties, dose, site of administration, and resulting local and systemic antimicrobial absorption), the choice and duration of systemic antimicrobial therapy, and the extent of surgical debridement. Nevertheless, randomisation attempts to distribute these contributing factors to treatment effect heterogeneity equally between the groups. This will contribute to the understanding of the validity of alternative systemic treatment strategies in the context of rising adoption of local antibiotic therapy for orthopaedic infection.

## Supplementary information

**Additional file 1.** Criteria for definite, possible and probable treatment failure; additional methodological description.

**Additional file 2.** SPIRIT 2013 Checklist: Recommended items to address in a clinical trial protocol and related documents.

## Data Availability

The full datasets generated during the current study will not be publicly available due to the potential for participant self-identification from the data but limited data that would not be identifiable may be published or sought from the senior authors. Reference to protocol items specified in the SPIRIT 2013 Checklist may be found in Additional file 2.

## Abbreviations

CDMS: Clinical Database Management System
DSMB: Data Safety and Monitoring Board
REC: Research Ethics Committee
SAE: Serious adverse event
TSC: Trial Steering Committee
