# Supplementary material for: Short or Long Antibiotic Regimes in Orthopaedics (SOLARIO): a randomised controlled open-label non-inferiority trial of duration of systemic antibiotics in adults with orthopaedic infection treated operatively with local antibiotic therapy
Source: Trials. 2019 Dec 9;20:693. doi: 10.1186/s13063-019-3832-3 (PMC6902346; doi:10.1186/s13063-019-3832-3)
Supplement: Supplementary file 1 — Additional file 1. Criteria for definite, possible and probable treatment failure; additional methodological description. [file 13063_2019_3832_MOESM1_ESM.docx]

**Supplementary methods**

**Rationale for choice of comparators**
It is not appropriate to withhold all systemic antibiotic therapy in patient undergoing surgery for bone infections. At least a short, perioperative course is required to act as prophylaxis against secondary infection acquired at surgery, to cover the inevitable bacteraemia caused by surgery and to provide adequate levels in the soft tissues round the surgical site, prior to elution of local antibiotic from the implanted carrier.

Studies reporting limited systemic antibiotic treatment alongside local antibiotic therapy describe systemic antibiotic therapy for up to 7 days following surgery.[1-5] Systemic antibiotic therapy for a period of up to 7 days following surgery allows sufficient time for microbiology analyses necessary for eligibility determination [6], and for patients to consider whether they wish to participate in the study.

**Additional antimicrobial therapy permitted within the stud**y

Patients will be permitted up to 7 consecutive days of systemic antimicrobial treatment, for the management of intercurrent infection unrelated to the incident orthopaedic infection without this being considered a protocol deviation. More than one such course of antibiotic therapy for an unrelated infection will be permitted without being considered a protocol deviation provided that there is a gap of at least 7 days in between courses.

Re-operation at the same anatomic site of infection will also be permitted but will, similarly, result in review by the blinded Endpoint Committee with respect to a possible recurrence of infection.

In the event of post-randomisation identification of micro-organisms fully resistant to the implanted local antibiotic therapy, the choice of further treatment will be deferred to the clinical care team in partnership with the patient; deviation from the protocol will be recorded in the event of prolonged or additional systemic antibiotic therapy. Similarly, if systemic antibiotic therapy is discontinued early as a result of side-effects or participant preference, this will also be recorded as protocol deviation.

**Definition of primary endpoint**

Definite treatment failure is defined by one or more of the following:

1. 1. The isolation by culture of micro-organisms from 2 or more samples of bone/peri-prosthetic tissue from the site of incident infection at re-operation, where the micro-organisms are phenotypically indistinguishable*.
2. 2. A pathogenic organism (e.g. *Staphylococcus aureus* but not *Staphylococcus epidermidis*) on a single, closed, biopsy or aspirate from the site of incident infection.
3. 3. Histology diagnostic of infection from bone/peri-prosthetic tissue obtained at re-operation at the site of incident infection.
4. 4. Formation of a sinus tract arising from the bone/prosthesis/orthopaedic device identified at clinical review or re-operation.
5. 5. Recurrence of frank pus adjacent to bone/prosthesis/orthopaedic device identified at re-operation or aspiration.
6. 6. Elevated synovial fluid White Cell Count or neutrophil percentage, or ++ change on leucocyte esterase strip, from prosthetic joint site affected by incident infection, at re-operation or aspiration.
7. 7. Death resulting from orthopaedic infection at the incident anatomic site.

**Definition of secondary endpoints**

Infection will be categorised by the blinded Endpoint Committee as “probable” where microbiological sampling had not been undertaken, AND none of the other criteria for definite infection had been fulfilled AND any one of the following were met:

1. The development of a discharging wound at the site of the bone/prosthetic joint/orthopaedic device at clinical review OR
2. Erythema, warmth and localised pain at the site of previously treated infection on clinical review OR
3. Radiological or operative findings of periosteal changes suggesting osteomyelitis or prosthetic joint infection OR
4. Rapid loosening of a joint prosthesis/orthopaedic device (i.e. leading to localised pain in less than 3 months since implantation), in the absence of a mechanical explanation for rapid loosening, on radiological, clinical or operative examination OR
5. The presence of deep pus close to but not adjacent to bone/prosthetic joint/orthopaedic device at reoperation OR
6. The presence of peri-prosthetic necrotic bone on operative histology.
7. Amputation at the site of initial orthopaedic infection.

Infection will be categorised as “possible” where microbiological sampling had been undertaken with negative results (according to criteria described above for “definite” infection) AND other criteria for definite infection were not fulfilled AND in addition one or more of the criteria listed a) to g) above was met. Proportions of participants in both treatment arms experiencing possible or probable treatment failure will be compared within the intention to treat and per-protocol populations.

* indistinguishable refers to the results of routine laboratory work, including bacterial genus/species and the results of routine antibiotic susceptibility testing. Additional bacterial typing in the laboratory beyond local routine practice is not required.

Where the blinded endpoint committee members differ in their initial categorization of a participant, consensus will be achieved by discussion or, if necessary, by majority opinion. The endpoint committee may, if necessary, take into account additional factors not listed above in the categorization of endpoints as definite, probable or possible.

**Validation of assessments and criteria for endpoints**

The choice of criteria for definite, probable and possible infection has been validated during a previous study of orthopaedic infection and has been updated in line with international guidance for the diagnosis of orthopaedic infection.[7-9] The blinded Endpoint Committee will be familiar these diagnostic criteria.

The EQ-5D-5L questionnaire is a validated instrument for assessment of quality of life, and has been used in prior studies of orthopaedic infection.[10]

Prospective enquiry and three level measurement of possible antibiotic side-effects follows the use of a similar tool in the assessment of symptoms associated with malignancy.[11] This method is expected to be more sensitive than adverse event reporting, which will also be used in this study.

**Additional secondary analyses**

Mortality will be compared between the two study arms using estimated hazard ratio with 95% confidence intervals.

A formal assessment of the difference in Serious Adverse Event frequency between the two treatment arms will be based on an estimated risk difference with 95% confidence intervals. The odds ratios of individual side-effects in both treatment arms will be compared using ordinal logistic regression.

Intravenous catheter-associated infection and Clostridium difficile-associated diarrhoea, diagnoses associated with systemic antibiotic treatment, will be analysed using logistic regression. Mean, median and distribution of the duration of hospital stay for participants in both treatment arms will be compared.

Quality of life will be analysed by considering absolute change scores from pre-operative baseline as well as population standardised values at each follow up time point. The corresponding distributions in each arm at each time point will be described by mean, median, 10^th^ and 90^th^ centiles, and visualised by violin plots. Formal assessment of the difference between the arms will be based on the value of the difference between pre-operative baseline and 12 month follow up EQ-5D-5L score; the difference in mean values and 95% confidence intervals for this will be reported.

The emergence and spread of antimicrobial resistance is still an unresolved problem in the hospital setting. One of the notable determinants of antimicrobial resistance is the selection pressure placed by the use of antimicrobials. As a result, the loss of antibacterial activity as a direct result of antibacterial consumption can be modelled via the economic approach of a negative externality. [12-14] The external (or indirect) cost of antibiotic use among study participants will be calculated using individual in-hospital antibiotic consumption and figures of the respective externality based on a literature search. In addition, the direct cost of hospitalization will be calculated using the patient’s length of hospital stay and standardised unit costs for the respective country.

**Creation of randomisation sequences**

Randomisation sequences for primary and back-up randomisation were created using the Blockrand[15] package for R[16] in Rstudio.[17] The randomisation module within the REDCap Clinical Database Management System[18] was used to assign consecutive treatment allocation based on the randomisation sequences. Creation, upload and deletion of sequences were performed blinded and supervised by an observer outside the study team.

**Back-up randomisation and allocation concealment**

Back-up opaque double envelopes containing consecutively-numbered randomisation cards, with treatment strategy assigned by permuted block randomisation, will be provided to each study centre for emergency use only. For verification of concealment, the inner envelope and card was produced in duplicate and assigned a unique number. Envelopes were created independently of the study team. Unused envelopes will be collected and checked against duplicates at the end of the study, to ensure they have been used consecutively and have not been tampered with.

**Study monitoring and interim analysis**

A trial steering committee (TSC) will be formed that will include an independent chair person, an independent deputy chair, two or more independent public/patient group representatives, the Chief Investigator, Principal Investigators and the trial coordinator. The TSC will meet at the beginning of the trial, after 100 patients have been recruited, and towards the end of the trial to *(i)* review protocol amendments and/or deviations, *(ii)* make recommendations regarding the conduct of the trial, and *(iii)* review recruitment and follow-up rates. Amendments to trial documents will be circulated to all participating centres.

A Data and Safety Monitoring Board (DSMB), comprising a senior statistician and two trialists with expertise in clinical infection, has met to discuss the study shortly before the start of the trial. Investigators will participate in this meeting. The DSMB will also meet prior to each TSC meeting, after 100 patients have been recruited, and at any other time they deem necessary to evaluate patient safety and frequency of endpoints in an un-blinded analysis. Investigators will not be present for discussion relating to un-blinded data. The DSMB may make recommendations to the TSC at any time during the trial.

**Serious Adverse Events**

All Serious Adverse Events (SAE) will be recorded in the e-Case Report Form. A Serious Adverse Event occurring to a participant will be reported to the Sponsor and the DSMB when, in the opinion of the Chief Investigator, the SAE is both unexpected and related to the allocated treatment arm.

**Criteria for discontinuation of the study**

When more than 100 participants have been recruited to the trial, a formal interim analysis of efficacy will be undertaken by the DSMB. It is expected that they will only recommend suspending the study if there is a very substantially worse outcome in one arm or concerns around patient safety in relation to the trial.

**Supplementary references**

[1] Buchholz HW, Elson RA, Engelbrecht E, Lodenkaemper H, Roettger J, Siegel A. Management of deep infection of total hip replacement. J Bone Joint Surg (Br). 1981; 63-B (3): 342.

[2] Calhoun JH, Henry SL, Anger DM, Cobos JA, Mader JT. The treatment of infected nonunions with gentamicin-polymethylmethacrylate antibiotic beads. Clin Orthop Relat Res. 1993; 295:23-27.

[3] Klemm, K. The use of antibiotic-containing bead chains in the treatment of chronic bone infections. Clin Microbiol Infect. 2001; 7:23-31

[4] Taggart T, Kerry RM, Norman P, Stockley I. The use of vancomycin-impregnated cement beads in the management of infection of prosthetic joints. J Bone Joint Surg [Br]. 2002; 84-B:70-2.

[5] Hoad-Reddick DA, Evans CR, Norman P, Stockley I. Is there a role for extended antibiotic therapy in a two-stage revision of the infected knee arthroplasty? J Bone Joint Surg [Br] 2004; 87-B: 171-4.

[6] Minassian AM, Newnham R, Kalimeris E, et al. Use of an automated blood culture system (BD BACTEC^TM^) for diagnosis of prosthetic joint infections: easy and fast. BMC Infect Dis. (2014):14:233

[7] Lew DP., and FA Waldvogel. Osteomyelitis. Lancet. 2004; 364(9431):369-7

[8] Osmon DR, Berbari EF, Berendt AR, Lew D, Zimmerli W, Steckelberg JM, et al., Diagnosis and management of prosthetic joint infection: clinical practice guidelines by the Infectious Diseases Society of America. Clin Infect Dis 2013; 56(1):1-10

[9] Parvizi J, and Gehrke T. Proceedings of the International Consensus Meeting on Periprosthetic Joint Infection. 2013. <http://www.msis-na.org/wp-content/themes/msis-temp/pdf/ism-periprosthetic-joint-information.pdf>

[10] Li HK, Rombach I, Zambellas R, Walker AS, McNally MA, Atkins BL, et al. Oral versus intravenous antibiotics for bone and joint infection. NEJM 2019; 380:425-36

[11] King MT, Viney R, Smith DP, Hossain I, Street D, Savage E, et al. Survival gains needed to offset persistent adverse treatment effects in localised prostate cancer. British Journal of Cancer. 2012:106:638 – 645

[12] Leal, J.R., J. Conly, E.A. Henderson and B.J. Manns (2017): How externalities impact an evaluation of strategies to prevent antimicrobial resistance in health care organizations, Antimicrobial Resistance & Infection Control 6:53.

[13] Kaier, K. (2012): Economic implications of the dynamic relationship between antibiotic use and hospital-acquired infections, Value in Health 15(1): 87-93.

[14] Kaier, K. and U. Frank (2010): Measuring the Externality of Antibacterial Use from Promoting Antimicrobial Resistance, Pharmacoeconomics 28(12): 1123-1128.

[15] Snow G. Package ‘blockrand’: Randomisation for block random clinical trials. Version 1.3 2015; https://cran.r-project.org/web//packages/blockrand/blockrand.pdf

[16] R Core Team. R: A language and environment for statistical computing. R Foundation for Statistical Computing, Vienna, Austria. 2013; http://www.R-project.org/.

[17] RStudio Team. RStudio: Integrated Development for R. RStudio, Inc., Boston, MA. 2015; <http://www.rstudio.com/>.

[18] Harris PA, Taylor R, Thielke R, Payne J, Gonzalez N, Conde JG. Research electronic data capture (REDCap) - A metadata-driven methodology and workflow process for providing translational research informatics support. J Biomed Inform. 2009; 42(2):377-81
